# Interleukin-25 as a Potential Biomarker in Lung Metastasis of Hepatocellular Carcinoma with HBV History in Chinese Patients: A Single Center, Case-control Study

**DOI:** 10.7150/ijms.90642

**Published:** 2024-05-19

**Authors:** Yuan Liao, Yixin Xu, Ziying Mo, Tianyi Zhu, Huimin Dong, Wenying Zhou, Qing Xia

**Affiliations:** 1 Department of Laboratory Medicine, the Third Affiliated Hospital of Sun Yat-sen University, Guangzhou 510630, China.; 2 School of Medicine, Shanghai Jiao Tong University, Shanghai 200025, China.; 3 Department of Oncology, State Key Laboratory of Oncogenes and Related Genes, Shanghai Cancer Institute, Renji Hospital, School of Medicine, Shanghai Jiao Tong University, Shanghai 200127, China.

**Keywords:** Interleukin-25, hepatocellular carcinoma, lung metastasis, prognosis

## Abstract

**Background:** Interleukin-25 (IL-25) has been proved to play a role in the pathogenesis and metastasis of Hepatocellular carcinoma (HCC), but the relationship between the level of IL-25 and the metastasis and prognosis of HCC is still not clear. This study aimed to investigate the expression of IL-25 and other potential biochemical indicators among healthy people, HBV-associated HCC patients without lung metastasis and HBV-associated HCC patients with lung metastasis.

**Methods:** From September 2019 to November 2021, 33 HCC patients without lung metastasis, 37 HCC patients with lung metastasis and 29 healthy controls were included in the study. IL-25 and other commonly used biochemical markers were measured to establish predictors of overall survival (OS) and progression-free survival (PFS) after treatment.

**Results:** The serum level of IL-25 was increased in HCC patients than healthy controls (*p* < 0.001) and HCC patients with lung metastasis had higher IL-25 level than HCC patients without metastasis (*p* = 0.035). Lung metastasis also indicated higher death rate (*p* < 0.001) by chi-square test, higher GGT level (*p* = 0.024) and higher AFP level (*p* = 0.049) by non-parametric test. Kaplan-Meier analysis demonstrated that IL-25 was negatively associated with PFS (*p* = 0.024). Multivariate Cox-regression analysis indicated IL-25 (*p* = 0.030) and GGT (*p* = 0.020) to be independent predictors of poorer PFS, while IL-25 showed no significant association with OS.

**Conclusion:** The level of IL-25 was significantly associated with disease progression and lung metastasis of HBV-associated HCC. The high expression of IL-25 predicted high recurrence rate and death probability of HCC patients after treatment. Therefore, IL-25 may be an effective predictor of prognosis in HCC.

## Introduction

Hepatocellular carcinoma (HCC) is one of the most common malignant cancers and the third leading cause of cancer-related death in China 1. HCC has a high mortality due to the occurrence of intrahepatic or systemic metastasis. AFP is the only biomarker that is most commonly used to predict the occurrence and prognosis of HCC patients, but its sensitivity and specificity are not satisfactory 2, 3. Thus, new and reliable biomarkers are needed to improve the diagnostic level of HCC. A few recent studies have suggested GGT as an independent prognostic indicator in cases with HCC. GGT was suggested to promote tumor progression and poor prognosis through inducing DNA damage and genome instability, releasing reactive oxygen species to activate invasion-related signaling pathways, blocking chemotherapy, regulating microRNAs, and managing CpG island methylation 4. A series of studies showed that the onset and progression of HCC was closely associated with the interaction between tumor cells and the inflammatory microenvironment 5. The inflammatory liver microenvironment also promotes the metastasis of HCC 6. Many studies reported that some inflammatory cytokines were included in the regulation of cancer cells, including IL-6 in breast cell and IL-20RB in bone metastasis of lung cancer 7, 8. IL-17 promoted the secretion of CAL27 cell and increased the infiltration of CD11c^+^ cells in the peritumoral area of basal cell carcinomas (BCCs) and squamous-cell carcinomas (SCCs) in mice 9.

HCC is prone to both intrahepatic and extrahepatic metastasis. Extrahepatic metastasis has been reported in 13.5 to 42% of HCC patients 10. Lung is the most common metastatic organ of primary liver cancer, accounting for 39.5%-53.8% of extrahepatic metastases. Lung metastasis seriously affects the prognosis of patients and is difficult to treat 11, 12. Inflammatory microenvironment has been proven to be crucial in HCC metastasis. Studied have shown that exosomal miR-1247-3p, which converts fibroblasts to cancer-associated fibroblasts (CAFs), could increase the secretion of IL-6 and IL-8 and promoted the lung metastasis of HCC 13. HIF-1α/IL-1β loop mediates the crosstalk between HCC cells and TAMs in a hypoxia-inflammatory microenvironment, which induces lung metastasis 14.

Interleukin-25 (IL-25, also called IL-17E) maps to chromosome 14q11 and it is an inflammatory IL-17 family cytokine. IL-25 was originally described as a Th2-produced cytokine involved in the induction of Th2-like responses 15. IL-25 has been proved to influence the promotion of nonmelanoma skin cancer, colorectal cancer and breast cancer 9, 16-18. Studies have showed that sustained innate IL-25 signaling helped to maintain the cancer permissive microenvironment of colorectal adenocarcinoma by preventing anti-tumor T cells and IFNγ-mediated immunity 17. Previous studies have demonstrated that IL-25 activate macrophages alternatively, secreted CXCL10 and activated the EMT pathway of HCC 19. However, there are few studies on the level of IL-25 in patients with lung metastasis of HCC and the prognosis on HCC.

This study was designed to determine the difference in IL-25 levels between HCC and HCC with lung metastasis and its association with the survival of patients with HBV-associated HCC.

## Materials and Methods

### Patients

From September 2019 to November 2021, a total of 99 patients were enrolled in the third affiliated hospital of Sun Yat-sen University, including 33 HCC patients without lung metastasis, 37 HCC patients with lung metastasis and 29 healthy controls (HC). The diagnosis of HCC was based on the diagnostic criteria for HCC used by the European Association for the Study of the Liver 20. Lung metastasis was diagnosed by chest computed tomography (CT) or magnetic resonance imaging (MRI). Patients with concurrent autoimmune disease or without history of HBV infection were excluded. All the patients underwent transcatheter arterial chemoembolization (TACE) as initial treatment.

### Clinical and laboratory data collection

The baseline data was taken within one week before treatment. Tumor location and TNM (tumor, lymph node and metastasis) staging were judged from the results of CT or MRI in accordance with the 7th edition of the AJCC Cancer Staging Manual 21. AST, ALT, GGT and TBA were included in serological detection. Serum levels of IL-25 were measured in HCC patient before treatment by ELISA (Cloud-Clone Corp. Wuhan, China) in accordance with the product instructions. Child-Pugh score was assessed in accordance with the 2021 NCCN guidelines 22. All consecutive parameters were categorized for further analysis as follows: age (≤65 or >65years), AFP (≤200 U/L or >200 U/L), nodule size (≤70cm or >70cm), AST (≤56U/L or >56U/L), ALT (≤40U/L or >40U/L), GGT (≤100U/L or >100U/L), PLT (≤130U/L or >130U/L). Cut-off values were set base on the previous studies (age 23, 24, tumor size 25 and AFP 26) or median of the data (AST, ALT, GGT, PLT).

### Follow‑up and outcome measures

US and computed tomography (CT) were conducted at one month after treatment and every 3-6 months thereafter. Extrahepatic organ examination was also carried out if patients had extrahepatic metastases. Liver magnetic resonance imaging was also used to define suspicious lesions demonstrated on CT. We defined progression as the appearance of new lesions with radiological features typical of HCC, as confirmed by at least two imaging methods for patients underwent curative treatment. For patients who underwent palliative treatment, progression was defined as at least a 20% increase in the sum of the longest diameter of the target lesions, or the appearance of new lesions or metastasis. The secondary outcome was overall survival (OS), which defined as the time from treatment to death for any reason.

### Statistical analysis

Mann-Whitney *U*-test was used to compare quantitative variables, and differences between categorical variables were analyzed by chi-squared or Fisher exact test. The associations between IL-25 and PFS as well as OS were evaluated. Univariate and multivariate Cox-regression analyses were done to discriminate clinicopathological predictors of PFS and OS, including group, age, gender, history of cirrhosis, nodule size and number, cancer embolus, number of metastases, TNM stage, AST, ALT, GGT, AFP, PLT, Child-Pugh score and IL-25. The differences of survival analysis were compared using the Kaplan-Meier method with log-rank tests for survival plot depiction. Hazard ratio (HR) and 95% confidence interval (95% CI) was evaluated. Only variables with statistical significance in univariate analysis were included in multivariate analysis.

Receiver operating characteristic (ROC) curves were depicted to set the cut-off points of IL-25. The classification variable was PFS. The reason for the choice of classification variables was that there were clinical trials demonstrating the association between IL-25 and PFS^2^. IL-25 was divided into high-level group and low-level group to investigate its relationship with other predictors.

The statistical significance level of all tests was *p<0.05*. Analyses and calculation were done by IBM SPSS Statistics 24.0.

## Results

### Patient characteristic

The demographics and characteristics of HCC patients were shown in Table 1. Among the 70 HCC patients in the study, 63 were male and 7 were female. The median age at baseline was 54.5 years (range, 25-81). During follow-up, tumor progression occurred in 67.1% of patients and 24.3% of patients died. All patients had a history of HBV infection and 91.4% had a history of cirrhosis. There were 31.4% of patients with a single primary nodule in liver, 37.1% with cancer embolus and 42.9% with more than 2 nodules of metastasis.

### Serum levels of IL-25 in HCC patients

**Figure 1:**
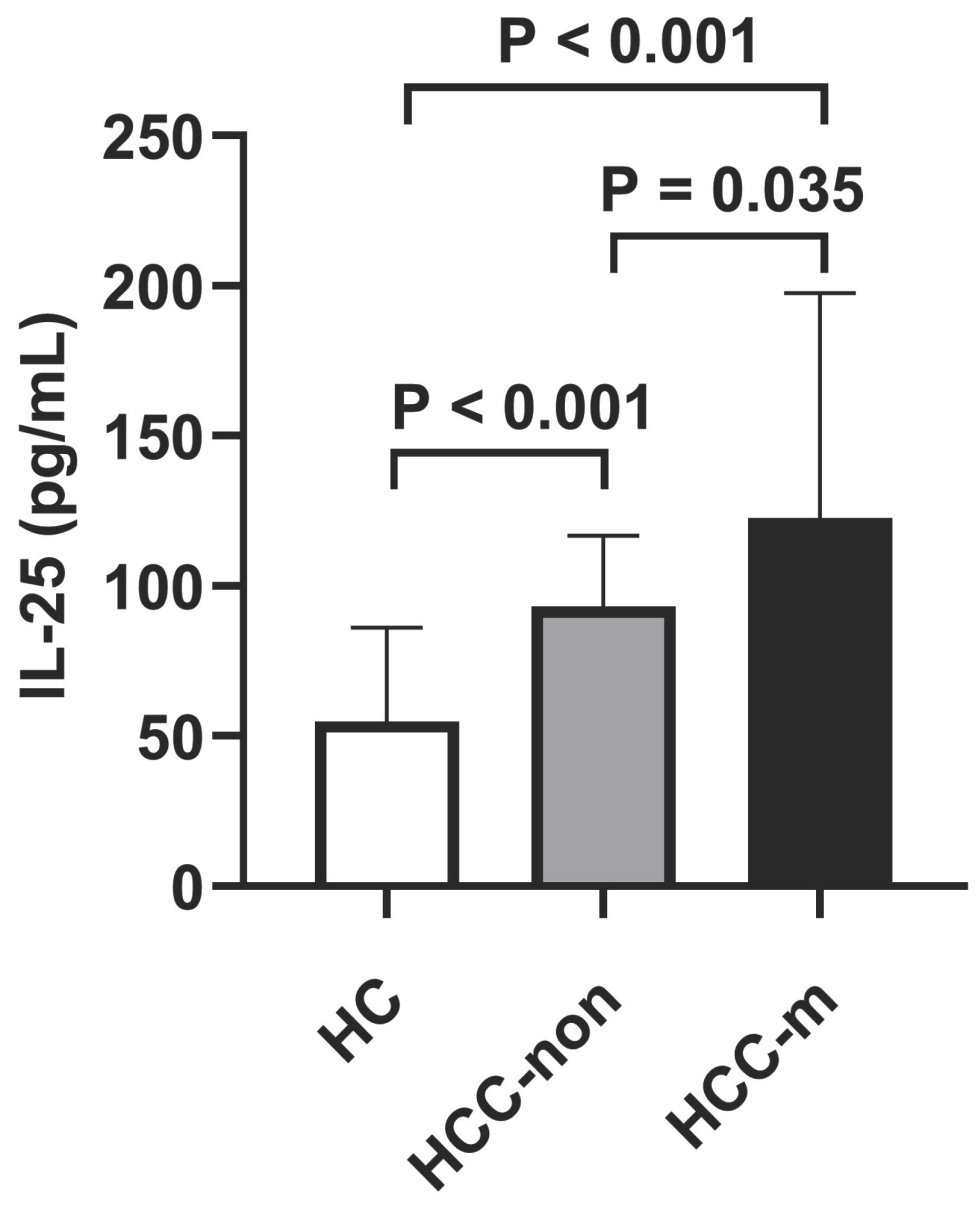
Levels of IL-25 in healthy control (HC) group vs non-metastatic HCC (HCC-non) group vs lung metastatic HCC (HCC-m) group. Bars represent the medians and interquartile range.

It was uncovered that HCC group suggested higher IL-25 levels than healthy control group (*p* < 0.001) and lung metastasis group suggested higher IL-25 levels than HCC group (*p* = 0.035, Fig. 1), which indicated that lung metastasis of HCC was significantly associated with IL-25. Lung metastasis also indicated higher death rate (*p* < 0.001), higher GGT (*p* = 0.024) and higher AFP (*p* = 0.049) (Table 1). Child-Pugh score also showed a marginal significance (*p* = 0.058).

### Effect of IL-25 on prognosis

**Figure 2:**
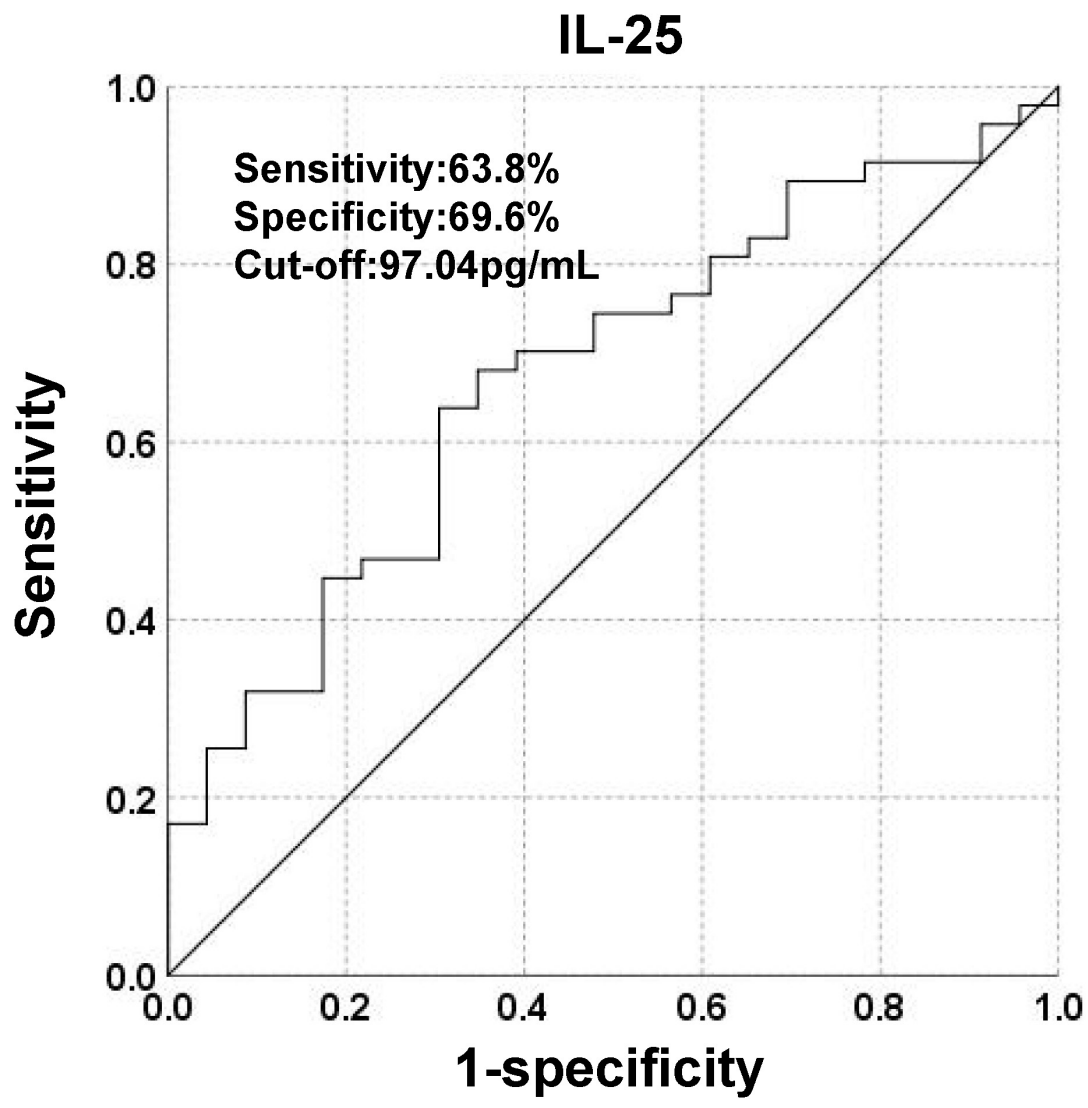
Receiver operating characteristic (ROC) curve analysis of IL-25 for the discrimination between progressed and non-progressed patients.

**Figure 3:**
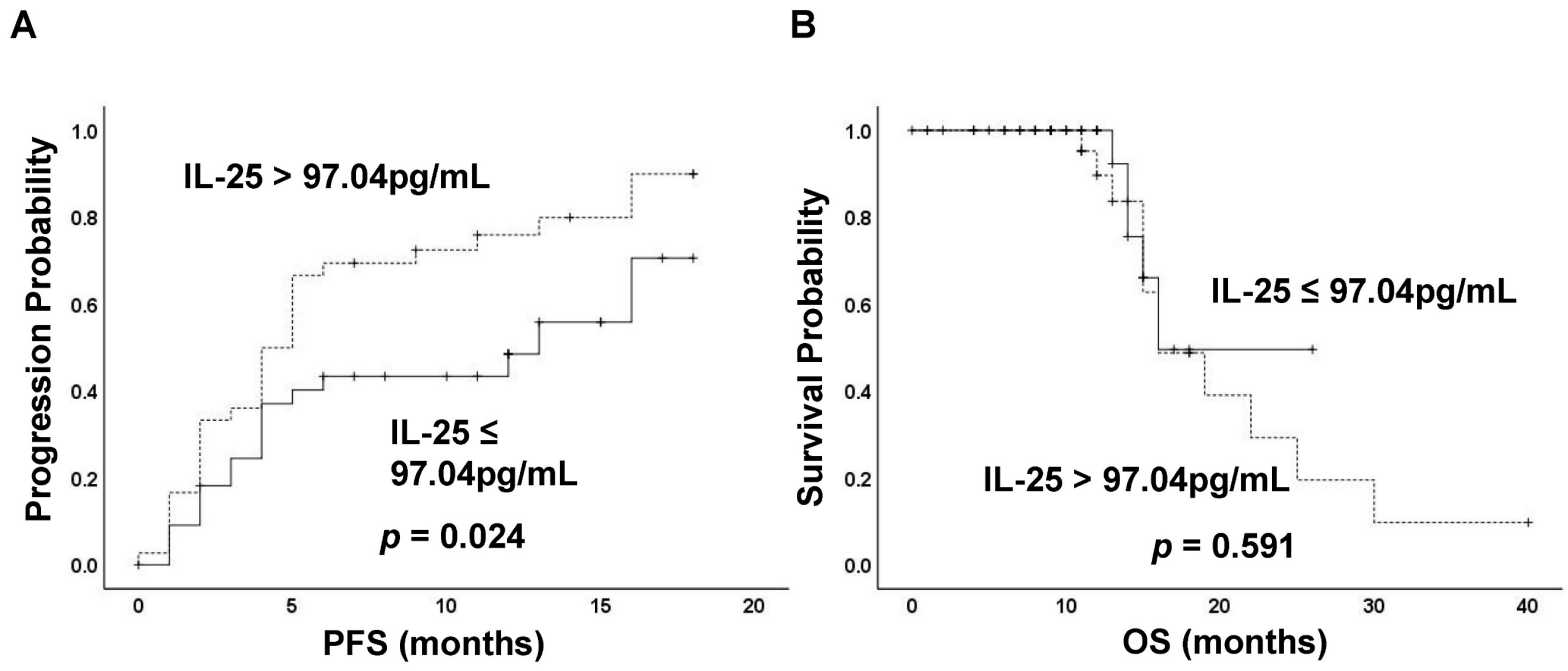
Kaplan-Meier curves stratified by the level of IL-25. (A) Progression-free survival (PFS) and (B) Overall survival (OS) of patients classified by IL-25 ≤ 97.04 pg/mL and > 97.04 pg/mL

To investigate the potential of IL-25 in predicting prognosis, a ROC curve was constructed to find the cut-off point of IL-25 based on tumor progression. The area under curve (AUC) was 0.675. A fixed cut-off value of 97.04 pg/mL was taken for the analysis, yielding a sensitivity of 63.8% and a specificity of 69.6% (Fig. 2). Kaplan-Meier analysis revealed that IL-25 level was significantly associated with PFS, with median PFS of 7 months and 4 months for patients with IL-25 ≤ 97.04 pg/mL and > 97.04 pg/mL respectively (*p* = 0.024) (Fig. 3A). However, IL-25 showed no association with OS. The median OS of patients with IL-25 ≤ 97.04 pg/mL was 12 months and that of patients with IL-25 > 97.04 pg/mL was 11 months (*p* = 0.591) (Fig. 3B). These results might indicate that HCC patients with higher levels of IL-25 tend to have worse prognosis.

To evaluate whether IL-25 was independent predictors of prognosis, univariate and multivariate cox-regression analyses for both PFS and OS were performed (Table 2). In the assessment of potential prognostic variables, GGT (*p* = 0.012) and IL-25 (*p* = 0.039) were found to be significantly associated with PFS by univariate cox-regression analysis. In addition, nodule size (*p* = 0.066) and AST (*p* = 0.056) showed marginally significant association with PFS in univariate analysis. At multivariate analysis, both GGT (*p* = 0.020) and IL-25 (*p* = 0.030) showed statistically significant association with PFS. In the assessment of OS, only cancer embolus showed marginally significant association in univariate cox-regression analysis (*p* = 0.084). IL-25 didn't show any association with OS in univariate analysis (*p* = 0.974).

### Associations between serum IL-25 levels and patient characteristics

With 97.04 pg/mL as the cut-off point, IL-25 was divided into high and low groups and the associations between IL-25 levels and clinicopathologic parameters were assessed by the chi-squared test (Table 3). Tumor size, PLT and serological indicators including AST, ALT and GGT were separated by the median of the data. High IL-25 level was found to correlate with high progression rate (*p* = 0.009) and more lung metastases nodes (*p* = 0.048). The death rate exhibits marginal significance with IL-25 (*p* = 0.079). However, we found no significant association between IL-25 and other clinical parameters including AST, ALT, GGT, AFP and PLT. IL-25 also had no association with cancer embolus, tumor size, TNM stage and Child-Pugh score.

## Discussion

IL-25 is an inflammatory IL-17 family cytokine that is confirmed to sustain type 2 immunity 15. Accumulated evidence indicates a key role of IL-25 in diseases including acute hepatitis, liver fibrosis and liver cirrhosis 2, 27. IL-25 was also confirmed to be associated with the onset and progression of various cancers, but it is rarely reported in the lung metastasis and the prognosis of HCC 28. In our study, we found that the serum level of IL-25 was significantly associated with the disease progression and lung metastasis of HBV-associated HCC.

Our results demonstrated that the serum level of IL-25 was increased in HCC patients than healthy controls and HCC patients with lung metastasis had higher IL-25 level than HCC patients without metastasis.

A tumor-infiltrating MMTV-PyMT tumor mouse model showed that tumor macrophages were the primary source of IL-25 and demonstrated the critical role of IL-25 in regulating the type 2 immune response by targeting Th2 cells in a breast cancer model, thereby promoting tumor metastasis 29. Another study confirmed that c-RAF phosphorylation, ERK1/2 and p70 S6 kinase induced by IL-17A and IL-17E were involved in tumor cell proliferation and survival 30. IL-25 can also induce JAK/STAT3 signaling pathway to promote self-renewal of cancer cells 31.

Some previous studies have initially uncovered the relationship between IL-25 and HCC. It was reported that the serum levels of IL-25 were significantly higher in patients with HCC than in patients with chronic hepatitis C and IL-25 was negatively correlated with serum zinc level, which promotes fibrosis of the liver 32. IL-25 could indirectly promote the progression of HCC cells and induce the alternative activation of macrophages to secrete CXCL 10 and activate the EMT pathway. It was uncovered that the alternative activation of macrophages induced by IL-25 promoted the migration, invasion and tumorigenesis of hepatoma cells, increased the expression of vimentin, Snail and phospho-ERK, and decreased the expression of E-cadherin in hepatoma cells 19. Moreover, another study also reported that IL-25 could predict the recurrence of patients with HBV-HCC after resection and help to diagnose HCC as a supplement to AFP 2. It indicated that IL-25 might be a potential tumor marker for the diagnosis of liver cancer. As a type 2 cytokine, IL-25 was also upregulated in many inflammatory disorders such as atopic dermatitis, psoriasis, and asthma 15. Thus, the specificity of IL-25 as a HCC biomarker was relatively low. In our study, we confirmed the relationship between HCC and IL-25 and further found that IL-25 provided independent prognostic information of prognosis in terms of PFS, whereas no significance in OS. It indicates that IL-25 may be a prognostic indicator of HCC progression and a potential target for gene therapy.

We also focused on the effect of IL-25 on HCC patients with lung metastasis. Lung metastasis is the most common distant invasive progression of HCC, and it is also one of the main causes of cancer-related death 33. The tumor microenvironment is a complex mixture, including various cytokines and stromal cells and extracellular matrix, which has important impacts on tumor metastasis 34. Recently, cancer associated fibroblast-derived CCL5 was reported to promote hepatocellular carcinoma metastasis through activating HIF1α/ZEB1 axis 35. Both *in vitro* and *in vivo* investigations identified that osteopontin had an important role in metastasis of HCC and was an attractive potential therapeutic target for combating HCC metastasis 36. Our study revealed that the increased expression of IL-25 was significantly associated with the lung metastasis of HBV-associated HCC, which indicates that IL-25 may be a predictor of lung metastasis in HCC.

We recognized several limitations in our research. The first was that it was a single-center retrospective analysis, which means a small sample size. Thus, the significance of multivariate cox-regression analysis was not high. The second was that no blood samples were taken during follow-up, thus the relationship between the change of IL-25 and the prognosis was not analyzed. The third was that more clinically proven HCC makers should be included to study their relationship with IL-25. Therefore, large-scale multi-center studies are warranted to validate and extend our results to confirm the clinical relevance of serum IL-25 as a prognostic biomarker in HBV-associated HCC patients in the future.

In conclusion, the serum level of IL-25 was significantly associated with the occurrence and lung metastasis of HBV-associated HCC and IL-25 may be an effective biomarker for HCC. The high expression of IL-25 had a negative effect on the progression probability of HCC patients after treatment, and a higher IL-25 level shortened the PFS time of HBV-associated HCC. Our findings suggest that IL-25 might be a promising independent outcome predictor and potential therapeutic target for HBV-associated HCC.

## Figures and Tables

**Table 1:** Patient characteristics

Variables	HCC without metastasis (n=33)	HCC with metastasis (n=37)	*p* value
Sex			0.565
Male (%)	30 (90.9)	33 (89.2)	
Female (%)	3 (9.1)	4 (10.8)	
Age, median (IQR), years	55 (27)	53 (17)	0.489
Progression			0.199
Present (%)	20 (60.6)	27 (73.0)	
Absent (%)	13 (39.4)	10 (27.0)	
Death			**< 0.001**
Present (%)	2 (6.1)	15 (40.5)	
Absent (%)	31 (93.9)	22 (59.5)	
Liver cirrhosis			0.607
Present (%)	30 (90.9)	34 (91.9)	
Absent (%)	3 (9.1)	3 (8.1)	
Tumor multiplicity			0.494
Single (%)	11 (33.3)	11 (29.7)	
Multiple (%)	22 (66.7)	19 (51.4)	
Cancer embolus			0.086
Present (%)	9 (27.3)	17 (45.9)	
Absent (%)	24 (72.7)	20 (54.1)	
TNM Stage			**< 0.001**
Ⅰ/Ⅱ/Ⅲ (%)	32 (97.0)	0 (0.0)	
Ⅳ (%)	1 (3.0)	37 (100.0)	
Child-Pugh score			0.058
≤6 (%)	13 (39.4)	7 (18.9)	
>6 (%)	15 (45.5)	23 (62.2)	
AFP (U/L)			**0.049**
≤200 (%)	17 (51.5)	12 (32.4)	
>200 (%)	13 (39.4)	24 (64.9)	
AST, median (IQR), U/L	54.0 (65.0)	61.0 (56.0)	0.441
ALT, median (IQR), U/L	39.0 (49.0)	39.5 (45.0)	0.746
GGT, median (IQR), U/L	61.50 (95.00)	94.5 (158.0)	**0.024**
PLT, median (IQR), ×10^9/L	130.5 (117.0)	122.5 (120.0)	0.430
IL-25, median (IQR), pg/mL	94.21 (52.27)	124.89 (121.19)	**0.035**

HCC, hepatocellular carcinoma; HC, healthy control; AFP, alpha fetoprotein; AST, aspartate transaminase; IQR, interquartile range; ALT, alanine aminotransferase; GGT, γ-glutamyl transpeptidase; PLT, platelet; IL-25, interleukin-25.

**Table 2:** Univariate and multivariate cox-regression analysis of factors associated with the progression and survival of HCC patients

Variables	Univariate analysis		Multivariate analysis
	PFS	OS	PFS
	HR (95% CI), *p*	HR (95% CI), *p*	HR (95% CI), *p*
Sex (female vs. male)	0.825 (0.326-2.091), 0.685	2.982 (0.628-14.168), 0.169	
Age (>65 vs. ≤65 years)	0.476 (0.202-1.124), 0.090	0.648 (0.084-5.014), 0.678	
Liver cirrhosis (present vs. absent)	1.870 (0.580-6.029), 0.295	1.113 (0.249-4.984), 0.889	
Tumor size (>7cm vs.≤7)	1.823 (0.962-3.456), 0.066	1.299 (0.266-6.348), 0.746	
Tumor multiplicity (multiple vs. single)	1.257 (0.648-2.440), 0.499	1.422 (0.417-4.846), 0.574	
Cancer embolus (present vs. absent)	1.335 (0.743-2.397), 0.334	2.495 (0.883-7.045), 0.084	
Number of metastases (>2 vs. ≤2)	1.177 (0.647-2.141), 0.593	0.403 (0.106-1.534), 0.183	
TNM stage (Ⅳ vs. Ⅰ-Ⅲ)	1.163 (0.636-2.129), 0.624	0.946 (0.102-8.782), 0.961	
AST (>56 vs. ≤56 U/L)	1.770 (0.986-3.175), 0.056	1.392 (0.423-4.575), 0.586	
ALT (>40 vs. ≤40 U/L)	1.248 (0.703-2.215), 0.449	1.031 (0.384-2.768), 0.952	
GGT (>80 vs. ≤80 U/L)	2.215 (1.189-4.127), 0.012	1.725 (0.455-6.546), 0.423	2.104 (1.127-3.928), **0.020**
AFP (>200 vs. ≤200 U/L)	1.460 (0.804-2.651), 0.214	1.241 (0.420-3.671), 0.696	
PLT (>130 vs.≤130 ×10^9/L)	1.007 (0.566-1.791), 0.981	1.596 (0.606-4.202), 0.344	
Child-Pugh score (>6 vs. ≤6)	1.090 (0.565-2.104), 0.798	0.821 (0.274-2.459), 0.724	
IL-25 (>97.04 vs. ≤97.04 pg/mL)	1.877 (1.033-3.411), 0.039	1.019 (0.327-3.175), 0.974	1.986 (1.069-3.688), **0.030**

Abbreviation: AFP, alpha fetoprotein; AST, aspartate transaminase; ALT, alanine aminotransferase; GGT, γ-glutamyl transpeptidase; PLT, platelet; IL-25, interleukin-25; PFS, progression-free survival; OS, overall survival.

**Table 3:** Associations between IL-25 levels and patients' characteristics

Characteristics	Low IL-25 group (n=33)	High IL-25 group (n=37)	*p* value
Gender			
Male	30 (90.9)	33 (89.2)	0.565
Female	3 (9.1)	4 (10.8)	
Age (yrs)			
≤65	27 (81.8)	28 (75.7)	0.371
>65	6 (18.2)	9 (24.3)	
Progression			
Present	17 (51.5)	30 (81.1)	**0.009**
Absent	16 (48.5)	7 (18.9)	
Death			
Present	5 (15.2)	12 (32.4)	0.079
Absent	28 (84.8)	25 (67.6)	
Liver cirrhosis			
Present	30 (90.9)	34 (91.9)	0.607
Absent	3 (9.1)	3 (8.1)	
Tumor size (cm)			
≤7	14 (42.4)	16 (43.2)	0.351
>7	17 (51.5)	14 (37.8)	
Tumor multiplicity			
Single	11 (33.3)	11 (29.7)	0.568
Multiple	21 (63.6)	20 (54.1)	
Cancer embolus			
Present	15 (45.5)	11 (29.7)	0.133
Absent	18 (54.5)	26 (70.3)	
Number of lung metastases nodes			
≤2	22 (66.7)	17 (45.9)	**0.048**
>2	10 (30.3)	20 (54.1)	
TNM stage			
Ⅰ-Ⅲ	18 (54.5)	14 (37.8)	0.123
Ⅳ	15 (45.5)	23 (62.2)	
AST (U/L)			
≤56	18 (54.5)	18 (48.6)	0.400
>56	15 (45.5)	19 (51.4)	
ALT (U/L)			
≤40	18 (54.5)	20 (54.1)	0.579
>40	15 (45.5)	17 (45.9)	
GGT (U/L)			
≤80	16 (48.5)	17 (45.9)	0.596
>80	15 (45.5)	16 (43.2)	
AFP (U/L)			
≤200	12 (36.4)	17 (45.9)	0.368
>200	18 (54.5)	19 (51.4)	
PLT (×10^9/L)			
≤130	17 (51.5)	18 (48.6)	0.500
>130	16 (48.5)	19 (51.4)	
Child-Pugh score			
≤6	10 (30.3)	10 (27.0)	0.457
>6	17 (51.5)	21 (56.8)	

Abbreviation: AFP, alpha fetoprotein; AST, aspartate transaminase; ALT, alanine aminotransferase; GGT, γ-glutamyl transpeptidase; PLT, platelet; IL-25, interleukin-25.
